# Supplementary material for: A PrEP decision aid for women survivors of intimate partner violence: Task-shifting implementation to domestic violence service settings
Source: PLoS One. 2024 Sep 17;19(9):e0310837. doi: 10.1371/journal.pone.0310837 (PMC11407614; doi:10.1371/journal.pone.0310837)
Supplement: S1 File — (DOCX) [file pone.0310837.s001.docx]

September 8, 2019

**APPROVAL OF SUBMISSION VIA EXPEDITED REVIEW**

**Approval Date**: 9/8/2019

**Expiration Date:** 10/1/2020

| **Investigator:** | Jaimie Meyer |
| --- | --- |
| **Type of Review:** | Continuing Review/Closure |
| **Title of Study:** | Project Options: Developing and Testing the Effect of a Patient-Centered HIV Prevention Decision Aid on PrEP uptake for Women with Substance Use in Treatment Settings |
| **IRB Protocol ID:** | 2000021561 |
| **Submission ID:** | CR00006180 |

Research activities associated with this submission are approved and may begin consistent with the terms of IRB approval.

The protocol continues to have benefits which outweigh the risks, deemed minimal.

See the next pages for important reminders and the list of IRB approved documents.

**Important Reminders:**

- By 8/2/2020, you are to submit documentation for a continuing review.
- You can submit a request to close research (end the IRB’s oversight) when:
  - - The protocol is permanently closed to enrollment,
    - All subjects have completed all protocol related interventions and interactions, and
    - Analysis of private identifiable information is completed.
- Changes must be submitted with a modification and approved by the IRB prior to implementation except to eliminate immediate hazards to participants. This includes changes to study procedures, informed consent documents, recruitment activities or study personnel.
- Information that requires prompt reporting to the IRB must be done so within 5 days of the PI becoming aware of the event (see Policy 710: Reporting Unanticipated Problems Involving Risks to Subjects or Others, including Adverse Events). This includes potential serious noncompliance, continuing noncompliance, and unanticipated problems to subjects or others.
- In conducting this activity, you should refer to and follow the Investigator Manual (HRP-103) as applicable, which can be found in the IRB Library within the IRB system.

**IRB REVIEW REFLECTS:**

• PrEP WAVE Aim 1_Stakeholder Compound Authorization 073119 .pdf, Category: Consent Form;

• Yale HIC protocol_updated 08072019, Category: IRB Protocol;

• PrEP WAVE Aim 1_Participant Compound Authorization - English 073119_.pdf, Category: Consent Form;

• PrEP WAVE Aim 2_Participant Compound Authorization - Spanish 073119 .pdf, Category: Consent Form;

• PrEP WAVE Aim 2_Participant Compound Authorization - English 073119 .pdf, Category: Consent Form;

• PrEP WAVE Aim 1_Participant Compound Authorization - Spanish 073119 .pdf, Category: Consent Form;

• Motherhood Focus Group Consent Form, Category: Consent Form;

Please keep this letter with your copy of the approved protocol documents.
