# Supplementary material for: A PrEP decision aid for women survivors of intimate partner violence: Task-shifting implementation to domestic violence service settings
Source: PLoS One. 2024 Sep 17;19(9):e0310837. doi: 10.1371/journal.pone.0310837 (PMC11407614; doi:10.1371/journal.pone.0310837)
Supplement: Attachment — Submitted filename: PLoS Response Letter_081924.docx [file pone.0310837.s003.docx]

August 19, 2024

Joel Msafiri Francis, MD, MS, PhD

Academic Editor, PLOS ONE

Dear Dr. Francise:

On behalf of our coauthors, thank you for your comments and ongoing consideration of our manuscript, “A PrEP Decision Aid for Women Survivors of Intimate Partner Violence: Task-Shifting Implementation to Domestic Violence Service Settings”. We revised the manuscript based on reviewers’ remarks, and all comments have been addressed as detailed below. All authors have seen and approved the manuscript for submission.

| **Reviewer Comments** | **Authors’ Response** |
| --- | --- |
| Please note that funding information should not appear in any section or other areas of your manuscript. We will only publish funding information present in the Funding Statement section of the online submission form. Please remove any funding-related text from the manuscript. | Funding information was removed from the manuscript text. |
| Please include your tables as part of your main manuscript and remove the individual files. Please note that supplementary tables (should remain/ be uploaded) as separate "Supporting Information" files | The Table was added to the main manuscript. |
